# Macrophage-Derived Extracellular Vesicles Induce Long-Lasting Immunity Against Hepatitis C Virus Which Is Blunted by Polyunsaturated Fatty Acids

**DOI:** 10.3389/fimmu.2018.00723

**Published:** 2018-04-12

**Authors:** Chengcong Cai, Benjamin Koch, Kenichi Morikawa, Goki Suda, Naoya Sakamoto, Sabrina Rueschenbaum, Sami Akhras, Julia Dietz, Eberhard Hildt, Stefan Zeuzem, Christoph Welsch, Christian M. Lange

**Affiliations:** ^1^Department of Medicine 1, Goethe University Hospital, Frankfurt am Main, Germany; ^2^Department of Medicine 3, Goethe University Hospital, Frankfurt am Main, Germany; ^3^Department of Gastroenterology and Hepatology, Hokkaido University Hospital, Hokkaido, Japan; ^4^Department of Virology, Paul-Ehrlich-Institut, Langen, Germany

**Keywords:** exosomes, interferon, innate immunity, omega-3 fatty acids, arachidonic acid

## Abstract

Extracellular vesicles (EVs) are increasingly recognized as important mediators of intercellular communication. In this study, we aimed to further characterize the role of macrophage-derived EVs in immune responses against hepatitis C virus (HCV) and the potential of polyunsaturated fatty acids (PUFAs) to modulate this modality of innate immunity. To this end, EVs were isolated from interferon-stimulated macrophage cultures or from serum of patients with acute or chronic hepatitis C. EVs were characterized by electron microscopy, flow cytometry, RNA-sequencing, and Western blot analysis. The effect of EVs on replication of HCV was assessed in coculture models. Functional analyses were performed to assess the impact of PUFAs on EV-mediated antiviral immunity. We found that macrophages secreted various cytokines shortly after stimulation with type I and II IFN, which orchestrated a fast but short-lasting antiviral state. This rapid innate immune answer was followed by the production of macrophage-derived EVs, which induced a late, but long-lasting inhibitory effect on HCV replication. Of note, exposure of macrophages to PUFAs, which are important regulators of immune responses, dampened EV-mediated antiviral immune responses. Finally, EVs from patients with hepatitis C exhibited long-lasting antiviral activities during IFN therapy as well. The antiviral effect of EVs from Caucasian and Japanese patients differed, which may be explained by different nutritional uptake of PUFAs. In conclusion, our data indicate that macrophage-derived EVs mediate long-lasting inhibitory effects on HCV replication, which may bridge the time until efficient adaptive immune responses are established, and which can be blunted by PUFAs.

## Introduction

Type I and II IFNs are key mediators of innate and adaptive immune responses in infectious diseases like hepatitis C or autoimmune diseases such as systemic lupus erythematosus (1, 2). Type I IFNs (e.g., IFN-α, IFN-β) and type II IFN (IFN-γ) signal through independent transmembrane receptors to induce Jak-dependent phosphorylation of signal transducer and activator of transcription (STAT) 1 and of STAT2. Type I IFN signaling predominantly results in heterodimerization of STAT1, STAT2, and IFN regulatory factor 9 to form the IFN-stimulated gene factor 3 (ISGF3) transcription factor, whereas type II IFN signaling mainly induces homodimerization of STAT1 (3). ISGF3 and p-Stat1 homodimers induce a large number of distinct, but overlapping ISGs, which together mediate an antiviral cellular state of virus-infected cells (4). Yet, after stimulation with type I or II IFNs, most cell types rapidly upregulate inhibitory mediators such as suppressor of cytokine signaling (SOCS) 1 or SOCS3, which results in refractoriness to further IFN-stimulation (2). Therefore, the question arises how innate immune responses control viral infections until fully adaptive immune responses are established.

Extracellular vesicles (EVs) are secreted by numerous cell types and contain a large variety of RNA species, proteins, and lipid mediators, which partially reflect the cellular content of the originating cell (5). EVs are not homogenous but rather consist of various subtypes which differ in size, vesicle content, and mechanisms of cellular release. Broadly, EVs can be classified into exosomes, which are 50–120 nm small, sediment at 100,000 × *g* during ultracentrifugation, and are released as intraluminal vesicles at the multivesicular body; and microvesicles, which are more heterogeneous, larger (50–1,500 nm), sediment at 10–14,000 × *g* and originate from direct budding at the plasma membrane (5). Convincing data have shown that EVs can merge with acceptor cells by membrane-mediated endocytosis and release functional RNA and protein mediators to the acceptor cells’ cytosol (6). Hence, membrane-sheltered transfer of parts of the cellular transcriptome, microRNAome and proteome offers unique opportunities of intercellular communication, potentially bypassing large distances within the body. Not surprisingly, there are indications that EVs contribute to various diseases including cancer, autoimmune diseases, infectious diseases, or various liver diseases (5, 7–11). In this regard, it was shown that liver sinusoidal epithelial cells and macrophage-like THP-1 cells are capable to secrete exosomes with suppressive effects on hepatitis B virus (HBV) and hepatitis C virus (HCV) (12, 13).

Free fatty acids, in particular omega-3 and omega-6 long-chain polyunsaturated fatty acids (PUFAs), have remarkable effects on innate and adaptive immune responses. In general, omega-3 PUFAs such as eicosapentaenoic acids (EPAs) and their metabolites are potent mediators of resolution of inflammation, whereas mediators derived from omega-6 fatty acids promote inflammation, pain, and coagulation (14, 15). In this study, we assessed the antiviral capacity of EVs from IFN-pulsed macrophages against HCV *in vitro* and *in vivo* and explored the impact of omega-3 and omega-6 PUFAs on this modality of innate immunity.

## Materials and Methods

### Cell Culture

Human monocytic THP-1 cells were cultured in RPMI (Gibco) supplemented with 10% heat-inactivated FBS and 1% penicillin–streptomycin. THP-1 cells were differentiated into macrophage-like cells using 50 nM phorbol 12-myristate 13-acetate (PMA) for 48 h. For the generation of human monocyte-derived macrophages (MDMs), buffy coats from healthy donors were used. Peripheral blood mononuclear cells (PBMCs) were separated by density gradient using Ficoll-Plaque Plus (GE Healthcare). CD14^+^ monocytes were isolated using CD14 microbeads (Miltenyi Biotec). Monocytes were cultivated in RPMI with 10% heat-inactivated FBS, 1% penicillin–streptomycin, and 20 ng/ml rhM-CSF (Peprotech) for 6 days to differentiate into MDMs. All the cells are maintained at 37°C in 5% CO_2_. Huh-7.5 human HCC cells were provided by Charles M. Rice (The Rockefeller University, New York, NY, USA). Subgenomic replicon construct pCon1/SG-Neo(I)/AflII (Con1 strain, genotype 1b) (16) was provided by Charles M. Rice. Huh-7.5 cells harboring subgenomic HCV replicons were cultured in DMEM supplemented with 10% heat-inactivated FBS, 1% penicillin–streptomycin, and 0.1% G418 (Carl Roth).

### Isolation of EVs From Cell Culture and From Patients With Hepatitis C

THP-1 cells or MDMs were washed three times with PBS, and the medium were changed to EV depleted medium (obtained by overnight centrifugation at 100,000 × *g*). EVs were isolated and purified from the cell culture supernatants using a differential centrifugation protocol (17). In detail, supernatants were collected, and cellular debris was removed by centrifugation at 300 × *g* for 10 min and 2,000 × *g* for 20 min. Subsequently, supernatants were collected and centrifuged again at 100,000 × *g* (SW28 rotor, Beckman Coulter) for 2 h. The pellet containing EVs was then washed in PBS, filtered through a 0.22 µm filter, and ultracentrifuged again at 100,000 × *g* for 2 h. The resulting pellets containing pure EVs were resuspended in 100 µl PBS and stored at −80°C or transferred directly to acceptor cells.

Extracellular vesicles from the serum of patients with acute or chronic hepatitis C or of healthy controls were purified using differential centrifugation. In detail, frozen serum was centrifuged at 2,000 × *g* for 20 min to remove aggregates. For exosome isolation, 500 µl of serum was loaded on top of a 10–40% iodixanol discontinuous gradient (18). After overnight 100,000 × *g* (Rotor SW28, Beckman Coulter) iodixanol gradient ultracentrifugation, exosome-enriched fractions were collected, washed in PBS, filtered through a 0.22 µm filter, and pelleted at 100,000 × *g* for 2 h. The pellet was resuspended in PBS. For isolation of microvesicles, 500 µl of serum was diluted with 1 ml PBS and centrifuged for 60 min at 10,000 × *g*. The supernatant was aspirated, and the pellet was resuspended in 1 ml PBS, centrifuged again for 60 min, and resuspended again in PBS.

All patients provided written informed consent to the study protocol, and the study was approved by the local ethic committees of the University Hospital Frankfurt, Germany, as well as by the Hokkaido University Hospital, Japan.

### Functional Assays

For transfer of whole cell culture supernatants, THP-1 cells or MDMs were pulsed with mock, IFN-α-2b (500 IU/ml, Roche), or IFN-γ (25 ng/ml, Peprotech) for 60 min. Then, the cells were washed three times with PBS, fresh media were added, and the supernatants were collected after 4–48 h. The supernatants were then centrifuged at 1,500 × *g* for 5 min and transferred to Huh-7.5 cells harboring subgenomic HCV genotype 1b (Con1) replicons. Alternatively, EVs were purified according to the above described protocol, and 100 µg (with respect to the protein amount) of purified EVs was transferred to Huh-7.5 cells harboring subgenomic HCV genotype 1b (Con1).

### Nanoparticle Tracking Analysis (NTA)

Extracellular vesicles were analyzed using a NanoSight NS500 instrument (Mavern). Particle movement was measured for 30 s and tracked by NTA software (version 2.3, NanoSight). Each sample was analyzed six times to get the mean, mode, and median vesicle size together with the vesicle concentration.

### Immunoblotting

Extracellular vesicles or whole cells were lysed by RIPA-Buffer (50 mM Tris, 150 mM NaCl, 0.1% SDS, 0.5% sodium deoxycholate, and 1% NP-40). Immunoblotting was performed as described previously (19) using the following antibodies: Antibodies against STAT1 p84/p91 (sc-346) was from Santa Cruz. Antibodies against p-STAT1 (Tyr701), p-STAT2 (Tyr689), and β-actin (AC-15) were from Cell Signaling, Millipore, and Sigma-Aldrich, respectively. Antibodies against CD81 (clone JS-81, BD Biosciences), Alix (clone 3A9, BioLegend), CD63 (clone H5C6, BioLegend), and TSG101 (clone C-2, Santa Cruz) were used to characterize EVs. Monoclonal antibody against HCV NS5A (9E10) was provided by Charles Rice, NY, USA.

### Cytokine Array

Supernatants from IFN or mock-pulsed MDMs were collected as described earlier. Cytokine analyses were performed according to the manufacturer’s instructions for use of the Proteome Profiler™ Human XL Cytokine Array Kit (R&D Systems). 102 different cytokines were detected and analyzed by the arrays.

### Transmission Electron Microscopy

Transmission electron microscopy was performed by negative staining according to standard procedure. 25 µl of the EVs was applied on carbon coated and glam discharged formvar grids. The grid was incubated with the sample at room temperature for 5–10 min. Then, the sample was socked out, and the grid was washed twice with 50 µl ddH_2_O. 15 µl of 2% uranyl acetate was then applied on the grid for 10 s at room temperature. The grid was dried at room temperature and scanned by TEM (EM 109, Zeiss, Jena, Germany).

### RNA Isolation and Quantitative Real-Time PCR (RT-PCR)

Total RNA from EVs or cultured cells was isolated using the RNeasy mini Kit (Qiagen). RNA concentration and quality were assessed with the Nanodrop 2000 (Thermo Scientific) and Agilent 2100 Bioanalyzer (Agilent), respectively. EV RNA was analyzed using RNA 6000 Pico kit (Agilent). CDNA synthesis was performed using the PrimeScript Reverse Transcription Kit (Takara) according to the manufacturer’s protocol. RT-PCR was performed using a QuantiTect SYBR Green PCR Master Mix (Qiagen) or Taqman Gene Expression Master Mix (Applied Biosystems). Primer sequences were as follows: GAPDH 5′-GAAGATGGTGATGGGATTTC-3′, 5′-GAAGGTGAAGGTCGGAGTC-3′; IFI44L 5′-GCTGCGGGCTGCAGAT-3′, 5′-CTCTCTCAATTGCACCAGTTTCC-3′; RSAD2 5′-CTTTGTGCTGCCCCTTGAG-3′, 5′-TCCATACCAGCTTCCTTAAGCAA-3′; ISG15 5′-TCCTGCTGGTGGTGGACAA-3′, 5′-TTGTTATTCCTCACCAGGATGCT-3′; IL28B 5′-GGCCTTTAAGAGGGCCAAAG-3′, 5′-GGCGGGAGCGGCACT-3′; GAPDH 5′-FAM-CCAGCCCCAGCAAGAGCACA-3′-TAM, 5′-CAAGGAGTAAGACCCCTGGA-3′, 5′-GGGTCTACATGGCAACTGTG-3′; HCV 5′-ACGCAGA-AAGCGTCTAGCCAT-3′; 5′-TACTCACCGGTTCCGCAGA-3′; 5′-FAM-TCCTGGAGGCTGCACGACACTCA-3′-TAM (Eurofins Genomics). Expression of mRNA species was expressed in relation to expression of GAPDH mRNA.

### RNA-Seq Library Preparation and Bioinformatics Analysis

60 ng of EV RNA was used as the starting input for RNA-Seq library preparation. RNA of EVs was sheared using a Covaris S2 Sonicater. Whole cDNA library was constructed using the TruSeq Stranded Total RNA Library Prep Kit (Illumina) with Ribo-Zero rRNA Removal Kit (Illumina). Amplified libraries were pooled and sequenced on a Nextseq 500 Sequencer (Illumina) for paired-read 75 cycles. For bioinformatics analysis, kallisto and sleuth with standard configurations were used. LncRNA transcripts and genes were annotated from NONCODE-database 2016 (http://www.noncode.org). The sequencing results were subsequently analyzed for Gene Ontology (GO) Term enrichment by DAVID (Database for Annotation, Visualization and Integrated Discovery v6.8) or for NONCODE GO terms by Blast2GO. Selected transcripts were confirmed by quantitative RT-PCR.

### Statistical Analyses

Statistical analyses were performed using BiAS, Version 11.06. Group differences were assessed by Wilcoxon–Mann–Whitney *U*-tests, as appropriate. Two-sided *P* values < 0.05 were considered to be statistically significant.

## Results

### Interferon-Pulsed Macrophages Rapidly Secrete Short-Acting Antiviral Mediators

**Figure 1:**
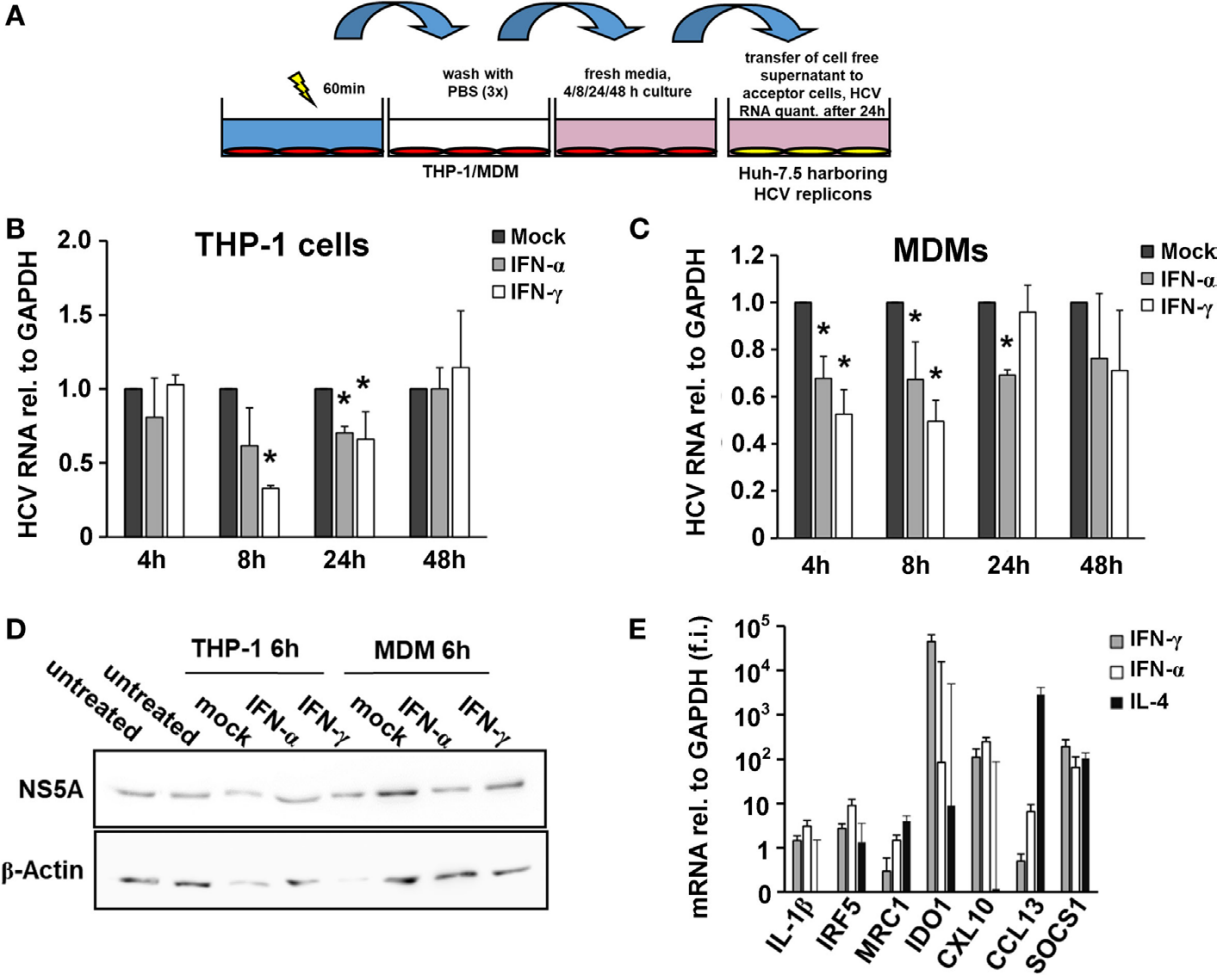
Interferon-pulsed macrophages inhibit hepatitis C virus (HCV) replication. **(A)** Schematic outline of the supernatant transfer experiment from macrophages to hepatoma cells harboring subgenomic HCV replicons. THP-1 cells were treated with 50 nM phorbol 12-myristate 13-acetate for 48 h to differentiate them into a macrophage-like phenotype. After 1 h of stimulation with mock, IFN-α (500 IU/ml) or IFN-γ (25 ng/ml), media were removed, and the cells were washed with PBS for three times to remove IFN entirely. Fresh media were added and collected after 4, 8, 24, and 48 h. Cell-free supernatant was transferred to hepatoma cells harboring subgenomic HCV replicons. After 24 h, cells were lysed for RNA isolation. **(B,C)** Relative HCV RNA replication levels of hepatoma cells harboring subgenomic HCV replicons after exposure to THP-1 **(B)** or MDM **(C)** supernatants. Maximal antiviral activities were observed 8 h after transfer of supernatants. Graphs show the mean and SE of triplicate samples taken at 4, 8, 24, and 48 h. RNA replication levels of mock-treated samples (*black bars*) were normalized to 1 and compared with IFN-α- (gray bars) or IFN-γ- (w*hite bars*) treated samples. **(D)** Western blot analyses of HCV NS5A protein levels of hepatoma cells harboring subgenomic HCV replicons after exposure to THP-1 or MDM supernatants. **(E)** Phenotyping of MDMs by quantitative PCR after treatment revealed polarization toward an M1 phenotype after stimulation with IFN. Quantities of mRNAs relative to GAPDH of IL-1β, IRF5, MRC1, IDO1, CXCL10, CCL13, and SOCS1 were assessed and expressed as fold induction (f.i.) relative to mock for M1/2 phenotypes. Graphs show the mean and SE of triplicate samples stimulated with IFN-α (*white bars*), IFN-γ (*gray bars*), or IL-4 (*black bars*) (*P < 0.05).

The monocytic leukemia cell line THP-1 is a widely used cell culture model to investigate macrophage biology. First, we differentiated THP-1 cells into a macrophage-like phenotype using PMA and pulsed them with type I or type II IFN for 4, 8, and 24 h. Supernatants of IFN-pulsed THP-1 cells were harvested and transferred to Huh-7.5 hepatoma cells harboring subgenomic HCV replicons (Figure 1A). As shown in Figure 1B, supernatants of IFN-pulsed THP-1 cells had antiviral effects with maximal antiviral activities observed after 8 h of IFN-treatment. To verify these findings in primary human macrophages, we isolated monocytes from PBMCs of healthy donors and differentiated them into MDMs. Phenotyping of MDMs by quantitative PCR revealed polarization toward an M1 phenotype after stimulation with IFN (Figure 1E). Supernatants of IFN-pulsed MDMs exhibited antiviral activities largely comparable, although even faster, to that of IFN-pulsed THP-1 cells (Figures 1C,D).

Next, we assessed whether EVs participate in this early antiviral immune response of macrophages. To this end, EVs were isolated from supernatants of IFN-pulsed MDMs. Antiviral activities of supernatants were not substantially reduced after depletion of EVs, and purified EVs had no relevant early antiviral activities (Figure S1 in Supplementary Material). Moreover, pretreatment with the exosome-inhibitor GW4869 did not affect the early antiviral activity of THP-1 cells after stimulation with IFN (Figure S1 in Supplementary Material).

**Figure 2:**
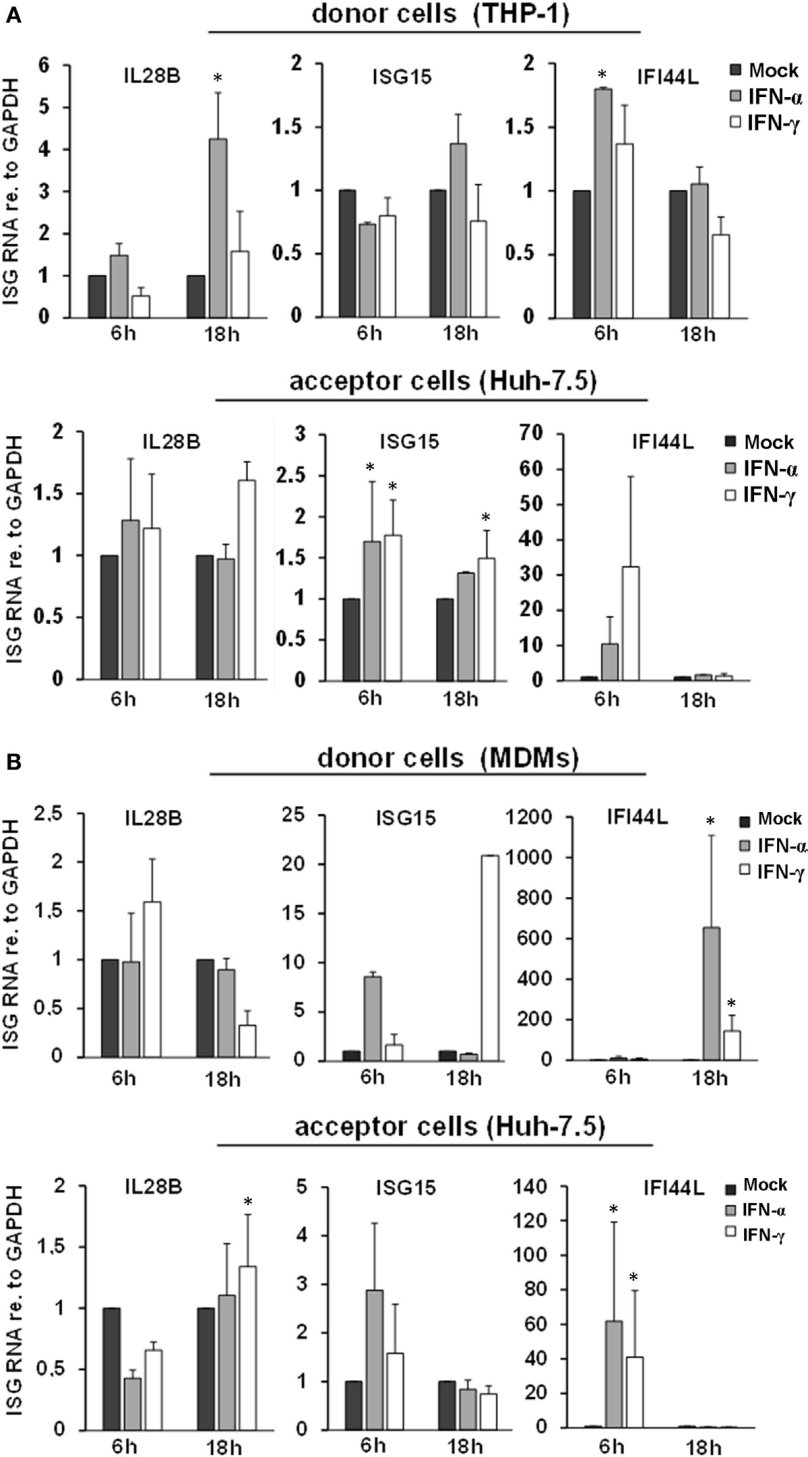
ISG induction and intercellular transfer from interferon-pulsed THP-1 cells **(A)** and MDMs **(B)**. Relative quantities of IL28B, ISG15, and IFI44L mRNAs were determined in THP-1 cells **(A)** and MDMs **(B)** (donor cells), as well as in hepatoma cells harboring subgenomic hepatitis C virus replicons (acceptor cells) by quantitative PCR. Donor cells were treated with mock, IFN-α (500 IU/ml), or IFN-γ (25 ng/ml) for 1 h and washed with PBS. After 6 and 18 h, the supernatant was transferred to acceptor cells, and mRNA was isolated from donor cells. 24 h after transfer, mRNA was isolated from acceptor cells. mRNA expression in mock-treated cells (*black bars*) was normalized to 1 and compared with IFN-α (*gray bars*) and IFN-γ (*white bars*) treated cells (*P < 0.05).

Since exosomal transfer of ISGs has been identified to transfer IFN-induced antiviral activity from macrophages to HBV-infected hepatocytes, we furthermore assessed ISG induction in THP-1 cells or MDMs (donor cells) and in Huh-7.5 cells harboring subgenomic HCV replicons (acceptor cells). A transient induction of ISGs was observed in MDMs, whereas in THP-1 cells only a marginal ISG induction to IFN-pulsation was observed (Figure 2). The latter finding may be explained by pre-activation of the endogenous IFN-system during the differentiation process of THP-1 cells, as in undifferentiated THP-1 cells ISG levels were lower at baseline but were profound induced by IFN-pulsing (Figure S2 in Supplementary Material). In acceptor cells, an inconsistent and only low ISG induction was observed after exposure to supernatants of macrophages/THP-1 cells, in particular in relation to ISG induction observed in donor and acceptor cells after stimulation of THP-1 cells with LPS as a reference inducer of IFN (Figure S3 in Supplementary Material). Taken together, our results do not support a role of EVs in this early phase of macrophage-mediated antiviral immunity. Instead, IFN-pulsed macrophages rapidly secrete a plethora of soluble factors such as cytokines or chemokines (Figure S4 in Supplementary Material), which may together orchestrate and early antiviral immune response.

### Interferon-Pulsed Macrophages Release EVs With Long-Lasting Antiviral Activity

**Figure 3:**
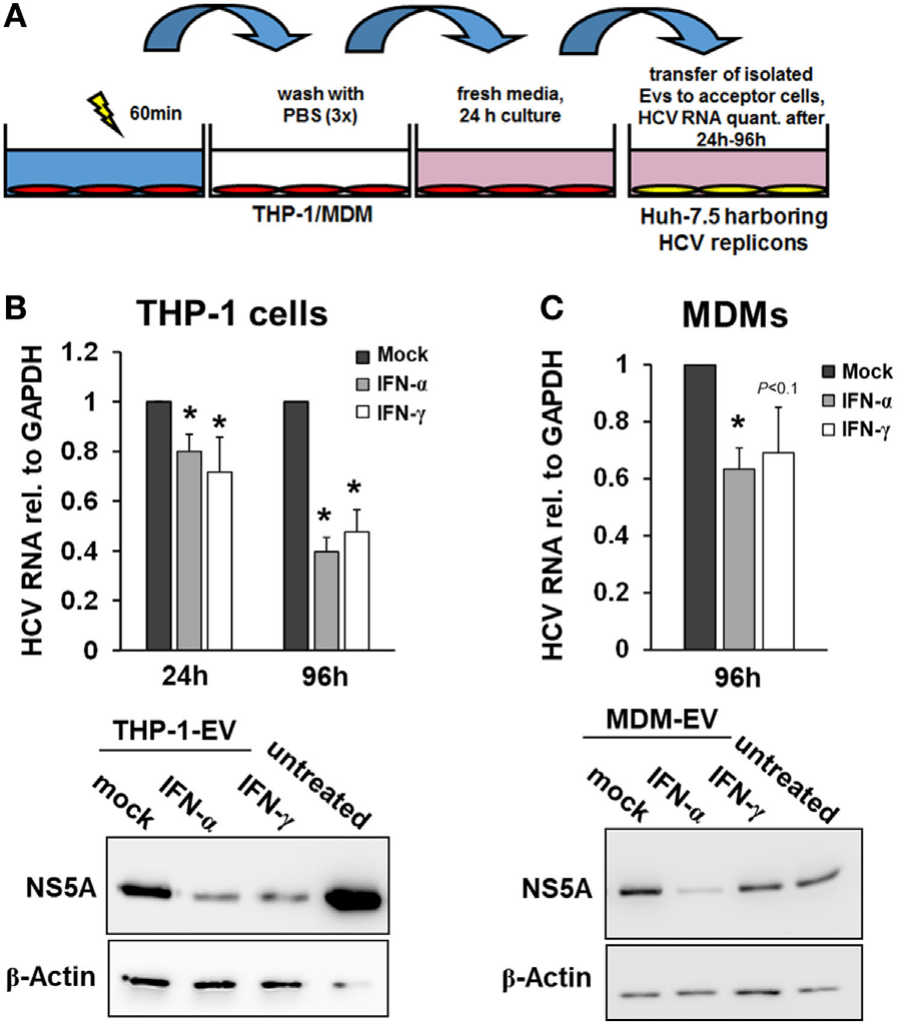
Macrophage-derived extracellular vesicles (EVs) inhibit hepatitis C virus (HCV) replication. **(A)** Schematic outline of EV transfer experiments. Macrophages were treated with mock, IFN-α (500 IU/ml), or IFN-γ (25 ng/ml) for 24 h. EVs were isolated from conditioned supernatants of macrophages using differential centrifugation. EVs containing 100 µg of protein were transferred to Huh-7.5 hepatoma cells harboring subgenomic HCV replicons. **(B,C)** EVs from IFN-pulsed THP-1 cells **(B)** and MDMs **(C)** inhibit HCV replication. HCV mRNA levels (upper panel) were quantified by Taqman real-time PCR 24 or 96 h after transfer of EVs. Mean and SEM of three independent experiments are shown. Western blot analyses of HCV NS5A protein levels after exposure to THP-1 or MDM supernatants for 96 h (lower panel) (*P < 0.05).

Since EVs did not participate in the early macrophage-mediated antiviral immune response, we next isolated EVs from THP-1 cells after 24 h of stimulation with IFN and transferred them to Huh-7.5 cells harboring subgenomic HCV replicons. Twenty-four hours after transfer of EVs, only a moderate decrease of HCV RNA levels was observed in cells exposed to EVs from IFN-pulsed versus mock-pulsed THP-1 cells. However, EVs of IFN-pulsed THP-1 cells resulted in a profound inhibition of HCV RNA replication after longer exposure times (72 h, not shown, and 96 h, Figure 3), compared with EVs from mock-treated THP-1 cells. EVs of IFN-pulsed MDMs had late suppressive effects on HCV RNA replication as well, though the antiviral activity of MDM-derived EVs varied remarkably between different donors (Figure 3).

### Characteristics of EVs of IFN-Pulsed THP-1 Cells and Primary MDMs

**Figure 4:**
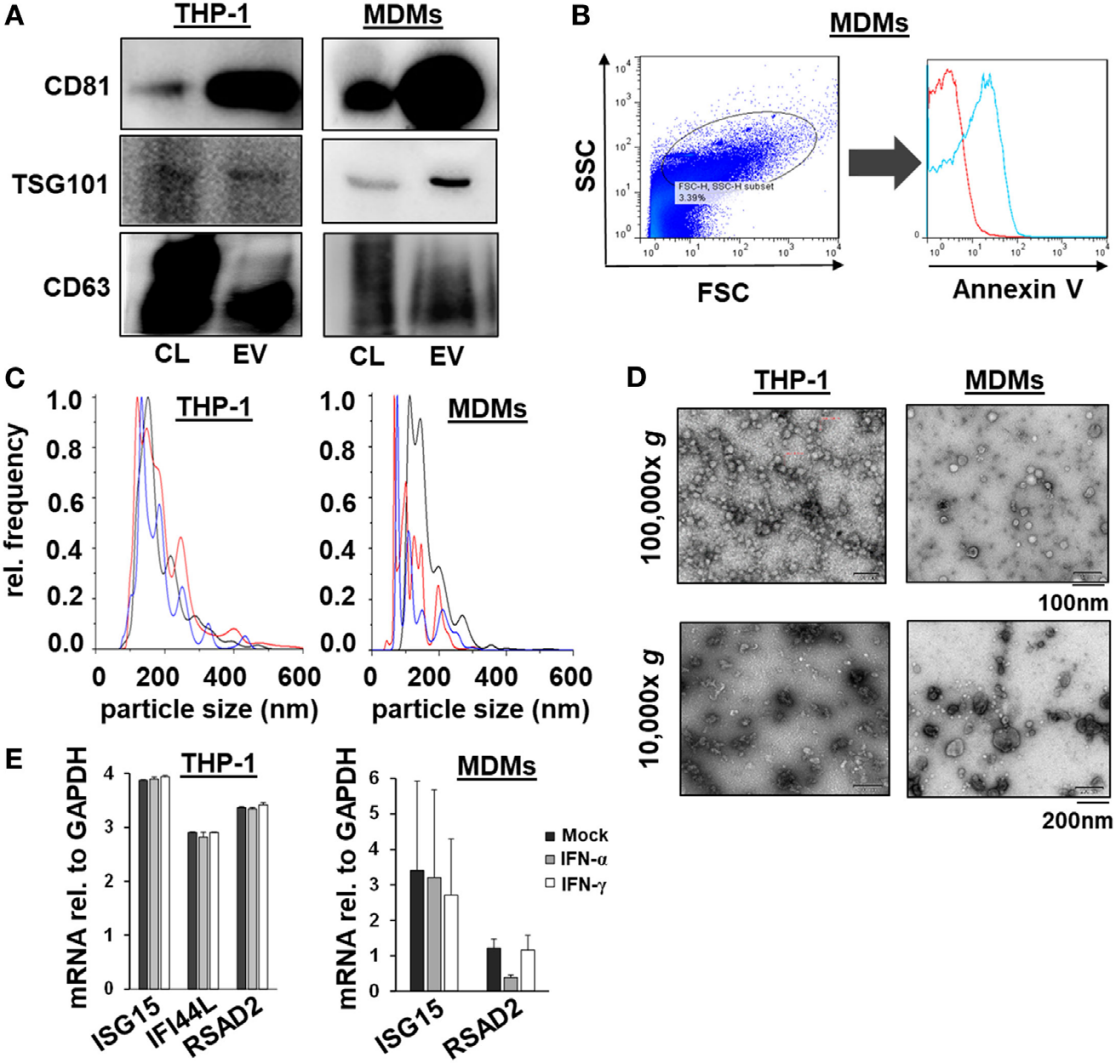
Characterization of macrophage-derived extracellular vesicles (EVs) by Western blot, flow cytometry, nanoparticle tracking analysis, electron microscopy, and quantitative PCR. **(A)** Western blots analysis of CD81, TSG101, and CD63 from EVs and cell lysates (CL) of naïveTHP-1 cells and MDMs (20 µg protein per lane). **(B)** Representative scatter blot and Annexin V staining of MDM-derived microvesicles mixed with calibration beads, as analyzed by flow cytometry (red unstained, blue Annexin V staining). **(C)** Averaged particle size distributions for EVs purified from naïve THP-1 cells and MDMs. Each colored line represents an independent technical replicate. **(D)** Electron microscopy of exosomes and microvesicles purified from naïve THP-1 cells and MDMs during differential ultracentrifugation. **(E)** Quantitative PCR analysis of mRNAs of selected ISGs (ISG15, IFI44L, and RSAD2) relative to GAPDH in EVs of mock (black bar), IFN-α (gray bar), and IFN-γ (white bar) treated macrophages; one representative experiment performed in triplicates is shown. IFI44L mRNA was inconsistently detected in EVs from MDMs and is therefore not shown.

**Figure 5:**
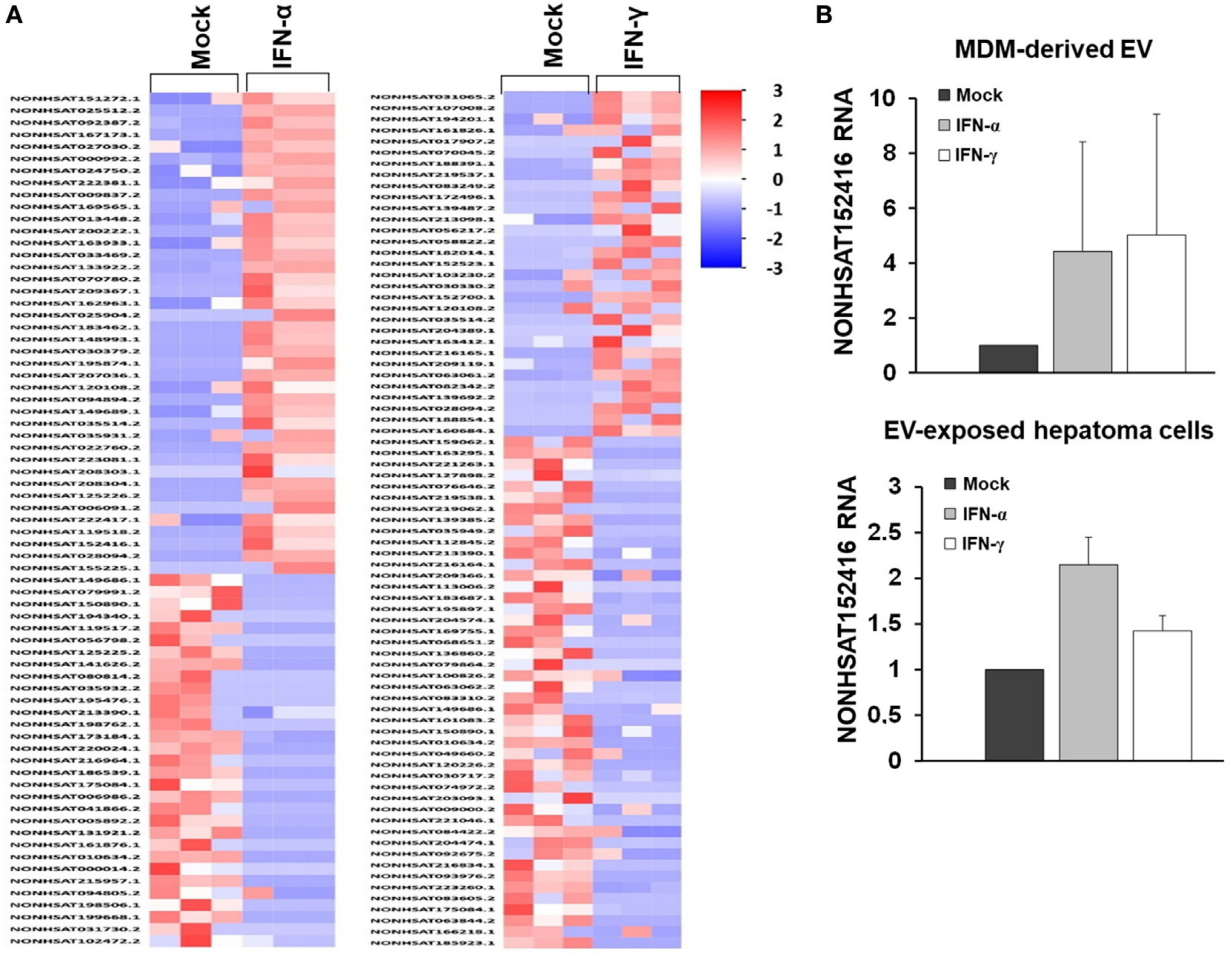
RNA sequencing of long-non coding RNA species of MDM-derived extracellular vesicles (EVs). **(A)** Heat map of long-non coding RNAs of EVs derived from three different donors. **(B)** Quantitative PCR of an exemplary long-non coding RNA (NONHSAT152416), relative to GAPDH, in MDM-derived EVs and in human hepatoma cells exposed to MDM-derived EVs (mean and SEM of three biological replicates are shown).

Given the comparable, but not equal effects of EVs derived from THP-1 cells and primary MDMs on HCV replication, we further characterized EVs from both cell types by electron microcopy, flow cytometry, and NTA. EVs isolated from supernatants of THP-1 cells 24 h after stimulation with IFN were rather homogenous and between 50–200 nm of size, whereas EVs from IFN-pulsed primary MDMs were more heterogeneous, partially larger (between 50 and 400 nm), and Annexin V positive in a significant fraction, the latter being a marker of microvesicles (Figure 4). Yet, there was no apparent difference in the content of marker proteins such as CD81 and ISG content between EVs derived from IFN-pulsed THP-1 cells or MDMs (Figure 4). Furthermore, no induction of selected classical ISGs was observed in EVs from THP-1 cells and MDMs upon stimulation with IFN (Figure 4). By contrast, RNA-sequencing of EVs of MDMs stimulated with type I or II IFN resulted in profound differences in the content of long-non coding RNAs of EVs, and EVs appeared to be able to transfer long-non coding RNAs to acceptor cells (Figure 5; Tables S1 and S2 in Supplementary Material); a phenomenon that has been shown previously for microRNAs (13).

### Polyunsaturated Free Fatty Acids Modulate the Antiviral Activity of EVs From IFN-Pulsed Macrophages

**Figure 6:**
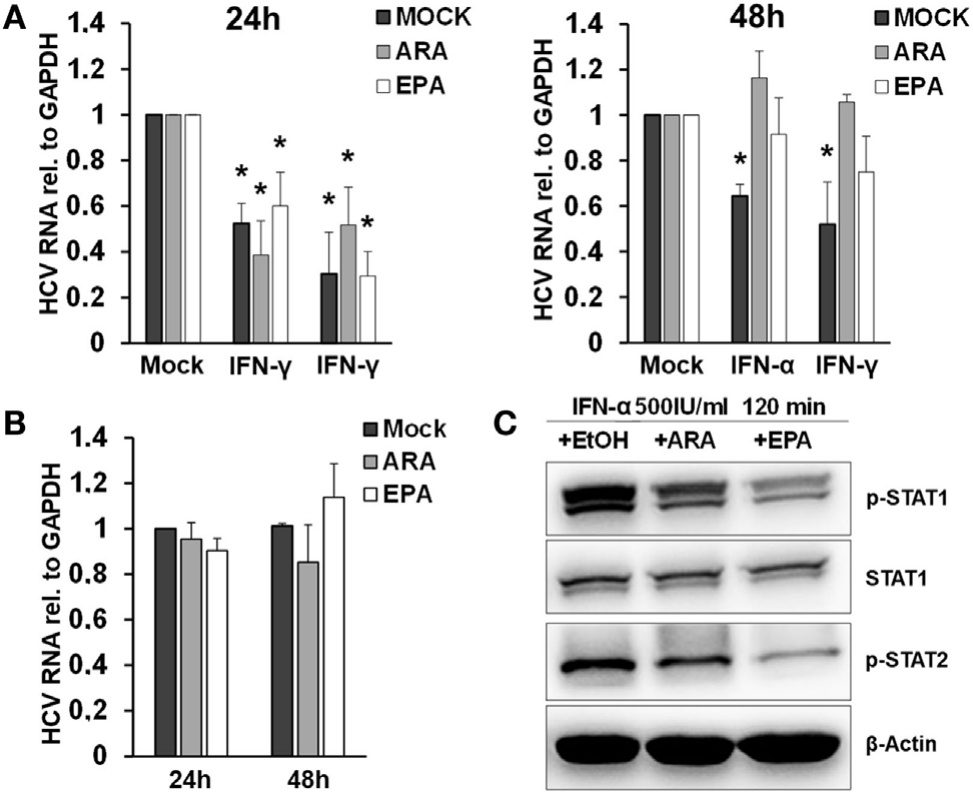
Polyunsaturated fatty acids modulate extracellular vesicle (EV)-mediated innate antiviral immunity. **(A)** THP-1 macrophages were treated pretreated with mock (black bar), arachidonic acid (ARA) (gray bar), and eicosapentaenoic acid (EPA) (white bar) for 24 h (left) and 48 h (right), respectively. Then, EVs were isolated from conditioned supernatants 24 h after pulsation with mock, IFN-α (500 IU/ml), or IFN-γ (25 ng/ml) for 1 h, respectively. Purified EVs containing 100 µg of protein were transferred to Huh-7.5 hepatoma cells harboring subgenomic hepatitis C virus (HCV) replicons. HCV mRNA levels were quantified by Taqman real-time PCR (RT-PCR) after 96 h. Mean and SEM of three biological replicates are shown. **(B)** Huh-7.5 hepatoma cells harboring subgenomic HCV replicons were exposed to mock (black bar), ARA (5 µg/ml, gray bar), or EPA (5 µg/ml, white bar) for 24 and 48 h, and HCV mRNA levels were quantified by Taqman RT-PCR. Mean and SEM of three biological replicates are shown. **(C)** Western blot analysis of p-STAT1 and p-STAT2 in cell lysates of THP-1 cells were treated with mock (EtOH), ARA (5 µg/ml), or EPA (5 µg/ml) for 48 h before stimulation with IFN-α (500 IU/ml) for 120 min (*P < 0.05).

Exposure of macrophages to free fatty acids has been shown to profoundly affect macrophage polarization and transcriptome (20). Moreover, it was shown that omega-3 PUFAs inhibit IFN signaling (21). Therefore, we hypothesized that exposure of macrophages to PUFAs may have an impact on the antiviral activity of macrophage-derived EVs. To test this hypothesis, we exposed THP-1 cells to arachidonic acids (ARAs) or EPAs, two key omega-6 and omega-3 PUFAs, respectively, for 24–48 h. Subsequently, THP-1 cells were pulsed with type I or type II IFN for 24 h, and EVs were isolated and transferred to Huh-7.5 cells harboring subgenomic replicons. Pretreatment of THP-1 cells for 48 h with both ARA and EPA before stimulation with IFN almost completely dampened the antiviral activity of THP-1 cell-derived EVs (Figure 6). Direct exposure of Huh-7.5 cells harboring subgenomic replicons to ARA or EPA did not affect HCV replication, suggesting a regulatory effect of ARA and EPA on THP-1 cells themselves. Since lipids might affect the production of membrane-derived EVs, we assessed quantity and size of EVs produced by THP-1 cells after stimulation with IFN. As shown in Figure S5 in Supplementary Material, a moderate reduction of the size of EVs produced by THP-1 cells after stimulation with IFN was observed in the presence of ARA and EPA, whereas ARA and EPA had no relevant effect on the quantity of EVs. However, Western blot analysis of p-STAT1 and p-STAT2 in IFN-stimulated THP-1 cells revealed impaired phosphorylation of these key mediators of IFN signaling in the presence of ARA and EPA (Figure 6).

#### EVs From Patients With Hepatitis C Inhibit HCV Replication During Treatment With Pegylated Interferon-Alfa and Ribavirin

**Figure 7:**
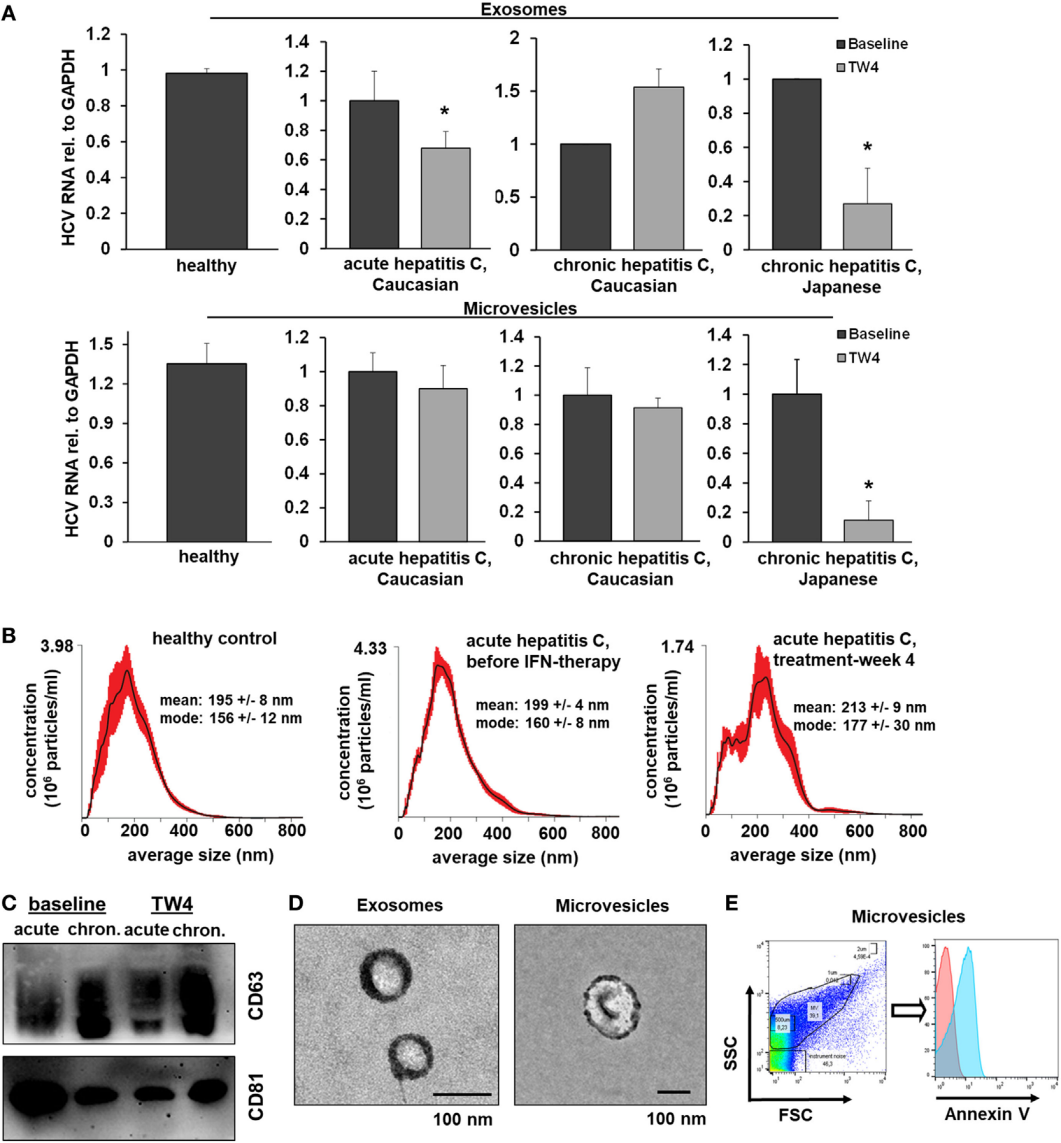
Extracellular vesicles (EVs) from patients with hepatitis C who are treated with PEG-IFN-α-based therapy inhibit hepatitis C virus (HCV) replication *in vitro*. **(A)** Exosomes (upper panel) and microvesicles (lower panel) were isolated from serum of healthy Caucasian donors or of Caucasian or Japanese patients with acute or chronic hepatitis C before and at treatment week 4 (TW4) of PEG-IFN-α-based antiviral therapy and transferred to human hepatoma cell lines harboring subgenomic HCV replicons. HCV RNA was quantified by quantitative PCR. Combined data of two independent experiments comprising EVs from three independent patients each are shown. Data are normalized to the baseline patients. **(B)** EV concentration and size distribution analysis of serum samples of patients with acute hepatitis C compared with healthy controls. **(C)** Western blot analysis of EV markers of patients with acute and chronic hepatitis C before and during antiviral therapy. **(D)** Electron microscopy of exosomes and microvesicles isolated from serum from a healthy control. **(E)** Representative scatter blot and Annexin V staining of microvesicles isolated from a healthy control mixed with calibration beads, as analyzed by flow cytometry (red-unstained, blue-Annexin V positive) (*P < 0.05).

To assess the *in vivo* relevance of EV-mediated antiviral immunity, we isolated EVs from serum of Caucasian patients with acute hepatitis C before and during antiviral therapy with pegylated (PEG)-IFN-α and ribavirin (no serum samples of Japanese patients with acute hepatitis C were available), Figure 7. Neither exosomes nor microvesicles from healthy controls inhibited HCV replication (Figure 7A). Furthermore, no relevant antiviral effect of exosomes or microvesicles from patients with acute hepatitis C was observed before the initiation of antiviral therapy (Figure 7A). However, exosomes (but not microvesicles) isolated from patients with acute hepatitis C at week 4 of treatment with PEG-IFN-α and ribavirin had a relevant inhibitory effect of HCV replication (Figure 7A). Next, we aimed to assess EVs from patients with chronic hepatitis C, who are much less sensitive to IFN therapy compared with patients with acute hepatitis C (22). As shown in Figure 7A, exosomes and microvesicles isolated from Japanese—but not from Caucasian—patients with chronic hepatitis C had a strong and long-lasting inhibitory effect on HCV replication during treatment with PEG-IFN-α-based antiviral therapy. It is well known that Japanese patients tend to have higher cure rates after therapy with PEG-IFN-α and ribavirin than Caucasian patients (22). One may speculate that these observed differences between Caucasian and Japanese patients may partially be explained by the well-established, profoundly lower nutritional uptake of omega-6 PUFAs (that abolished EV-mediated antiviral immunity *in vitro*) in the Japanese compared with the Caucasian population—an hypothesis which, however, we could not assess experimentally in our cohort (23). The quality of serum-derived exosomes and microvesicles was confirmed by size distribution analysis, electron microscopy, flow cytometry, and Western blot analysis (Figures 7B–E).

## Discussion

Our study reveals a biphasic model of IFN-induced macrophage-mediated antiviral immunity. In detail, our data show that IFN-pulsed macrophages secrete soluble mediators within hours after IFN-exposure, which inhibit HCV replication independently from the production of EVs. This rapid, but short-lasting immune response is followed by secretion of EVs, which orchestrate an antiviral cellular state that evolves during days in virus-infected acceptor cells. Of note, the release of EVs with antiviral properties is blunted by exposure of macrophages to PUFAs by inhibition of IFN-induced JAK–STAT signaling.

Here, described biphasic model of macrophage-mediated antiviral immunity, in which EVs exert a long-lasting antiviral state after a single IFN-pulse, may provide a mechanism, which allows the innate immune system to bridge the gap between the early IFN response that involves the secretion of soluble mediators from macrophages as well as direct IFN-induced ISG induction in hepatocytes, and the successful establishment of adaptive immune responses, which are required for definite viral clearance (24–26). In particular, long-lasting EV-mediated virus control may help to overcome detrimental effects of IFN refractoriness, which occurs within hours after stimulation of virus-infected cells with IFN (2). In this regard, our finding that EVs from patients during PEG-IFN-α-based therapy exhibit long-lasting antiviral immunity further supports the notion that once-weekly administered PEG-IFN-α inhibits HCV replication *in vivo*, despite of only transient activation of the JAK–STAT pathway and immediate and long-lasting upregulation of negative regulators such as USP18 (27).

Extracellular vesicles are increasingly recognized as components of the immune response against hepatotropic (and other) viruses. Recent studies have shown that THP-1 cells (no primary macrophages were used in this study) and liver sinusoidal endothelial cells secrete exosomes that inhibit HBV and HCV replication by transfer of a cocktail of antiviral mRNAs and microRNAs after stimulation with IFN-α, respectively (12, 28). In this context, it appears important that in our study classical ISGs were likely not responsible for EV-mediated antiviral immunity. Neither, relevant differences of classical ISGs in EVs (and in acceptor cells) from IFN-pulsed versus control macrophages were observed nor would the late antiviral effect of EV-mediated antiviral immunity fit to rapid effects mediated by classical ISG. Therefore, it appears likely that the antiviral activity of macrophage-derived EVs is rather mediated by mediators, which impact on regulation of acceptor cell gene expression. Indeed, it is known that IFN induces specific signatures of microRNAs in exosomes (13), and we have observed remarkable differences in the composition of long-non coding RNAs in EVs from MDMs stimulated with IFN compared with controls. Yet, the detailed mechanism of EV-mediated antiviral immunity remains to be addressed in future studies.

Our study has revealed that primary human macrophages release smaller numbers, but larger and more heterogeneous EVs compared with those derived from THP-1 cells. Nevertheless, we could show that EVs from primary macrophages and EVs isolated directly *ex vivo* from patients with hepatitis C exert relevant antiviral activities during IFN therapy, suggesting *in vivo* relevance of our findings. Our data support the recently published expert opinion that a better understanding of EVs derived from primary cells is required to fully understand the biological relevance of EV-mediated signaling (29).

Long-chain PUFAs are important mediators of inflammation, adaptive immune responses, and resolution of inflammation. Omega-3 and omega-6 PUFAs predominantly promote pro-resolution and pro-inflammatory effects, as they are precursors of resolvins/protectins and prostaglandins/leukotrienes, respectively (14, 15). Yet, there is increasing evidence that omega-3 and -6 PUFAs exhibit direct biological functions as well. For example, a recent study has shown that EPA and DHA inhibit the NLRP3 inflammasome in macrophages, independently from their enzymatic products (30). Our finding that exposure of macrophages to both omega-3 and omega-6 PUFAs blunts the release of EVs with antiviral properties (presumably *via* blunting innate immune induction of macrophages by IFN) suggests a rather direct effect of long-chain PUFAs as well. Indeed, we could show that ARA and EPA prevent IFN-induced phosphorylation of STAT1 and STAT2, two key transcription factors of type I and II IFN signaling, which is a likely mechanistic basis of our findings. Our findings are in line with previous studies showing that omega-3 and -6 PUFAs blunt IFN-γ signaling in peritoneal macrophages from mice infected with *Listeria monocytogenes* as well as IFN-induced IL-18 binding protein expression in prostate cancer cells (21, 31).

It is important to note that the concentration of omega-6 PUFAs outweighs that of omega-3 PUFAs in human tissues and blood manifolds (32). Hence, the inhibitory effect of PUFAs on EV-mediated antiviral immunity might be predominantly driven by omega-6 PUFAs *in vivo*, an assumption which would fit to the remarkable differences in the antiviral effect of EVs from Japanese versus Caucasian patients. Differences in nutritional habits between Japanese and Caucasian populations are well known, which are characterized in particular by low and high uptake of omega-6 PUFAs, respectively (23). However, it is important to highlight that this hypothesis remains speculative since we did not have information on nutrition habits of our patients and failed to quantify PUFAs by mass spectrometry in our patients. Additional factors such as the genetic background of the different patient cohorts may provide other explanations for the observed differences between Caucasian and Japanese patients.

Our study has several limitations. First of all, the mechanism of the different effects of EVs from Japanese versus European patients remains speculative. Furthermore, the molecular mechanism of EV-mediated antiviral immunity remains to be elucidated. Finally, our study did not address the effect of EVs on other steps of the HCV life cycle than HCV replication.

In conclusion, our findings identify macrophage-derived EVs as long-lasting mediators of antiviral immunity, potentially bridging the gap of early IFN signaling until sufficient adaptive immune responses are established. High uptake of omega-6 PUFAs may dampen this modality of antiviral immunity *in vivo*.

## Ethics Statement

The study was approved by the local ethical committee of the Goethe University Hospital Frankfurt as well as of the Hokkaido University Hospital.

## Author Contributions

The authors, CC and CML have contributed to the manuscript by planning the study. CC, BK, KM, SR, SA, JD, MU, EH, SZ, CW, and CML have contributed by collecting the data. CC, BK, SR, SA, JD, MU, EH, SZ, CW, and CML have contributed by analyzing the data. The Co-authors, GS and NS have contributed to collecting the data. CML and CC have drafted the manuscript, and all the authors have revised the manuscript.

## Conflict of Interest Statement

CML: unrestricted research support from Gilead Sciences. SZ: receiving lecture fees and consulting fees from AbbVie, Bristol-Myers Squibb, Gilead, Janssen, and Merck. The other authors have no conflicts of interest to report.
