# Supplementary material for: Macrophage-Derived Extracellular Vesicles Induce Long-Lasting Immunity Against Hepatitis C Virus Which Is Blunted by Polyunsaturated Fatty Acids
Source: Front Immunol. 2018 Apr 12;9:723. doi: 10.3389/fimmu.2018.00723 (PMC5906748; doi:10.3389/fimmu.2018.00723)
Supplement: Supplementary file 3 [file Data_Sheet_1.PDF]

SI Figures

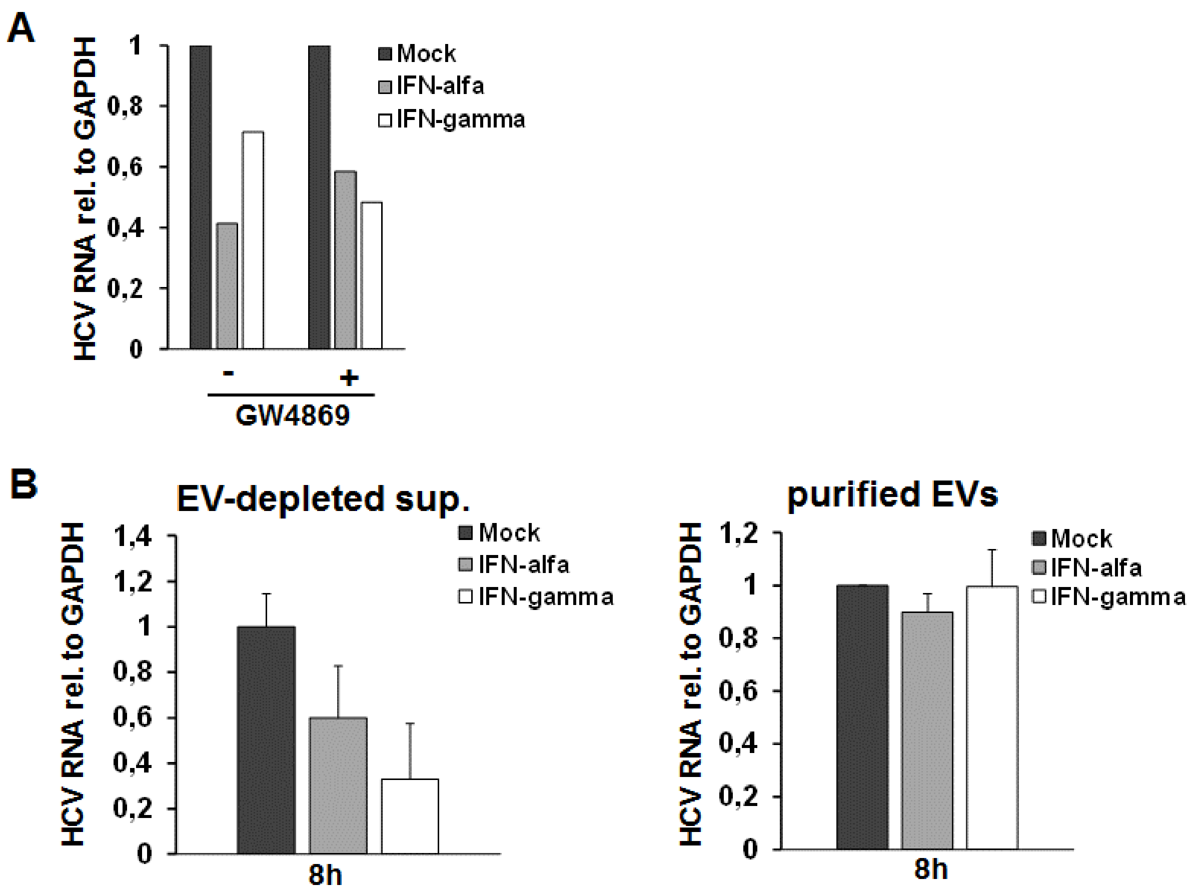

SI Figure 1. Extracellular vesicles are not involved in early macrophage-mediated antiviral immunity. (A) Relative HCV RNA levels in Huh-7.5 cells harbouring subgenomic HCV replicons after exposure to supernatants of THP-1 cells. THP-1 cells were pre-treated with the exosome inhibitor GW4869 (250nM) or vehicle for 48h prior to stimulation with mock (black bar), IFN-α (500IU/ml, grey bar) or IFN-γ (25ng/ml, white bar) for 1h. Supernatants were harvested after 8h. (B) Relative HCV RNA levels in Huh-7.5 cells harbouring subgenomic HCV replicons after exposure to EV-depleted supernatants *versus* purified EVs of THP-1 cells. THP-1 cells were stimulated with mock (black bar), IFN-α (500IU/ml, grey bar) or IFN-γ (25ng/ml, white bar) for 1h. Supernatants were harvested after 8h.

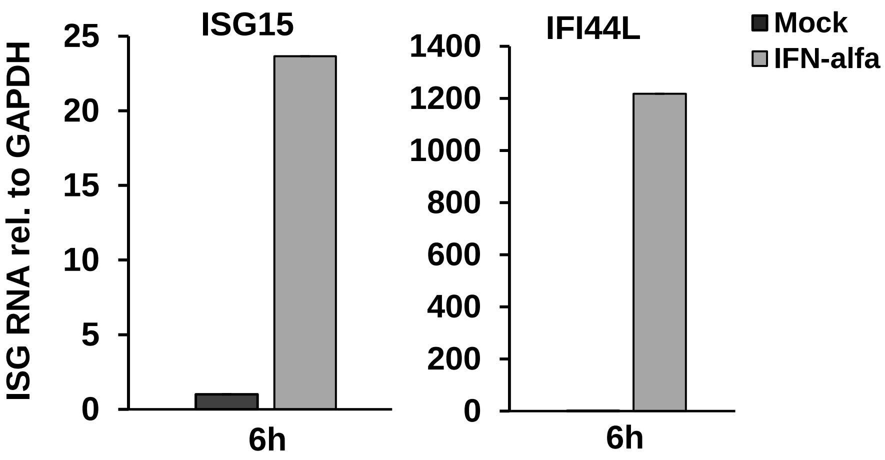

Figure 2: ISG induction in undifferentiated THP1 cells. Relative mRNA levels of ISG15 and IFI44L in undifferentiated THP-1 cells after stimulation with mock (black bar) or IFN-α (500IU/ml, grey bar) for 6h.

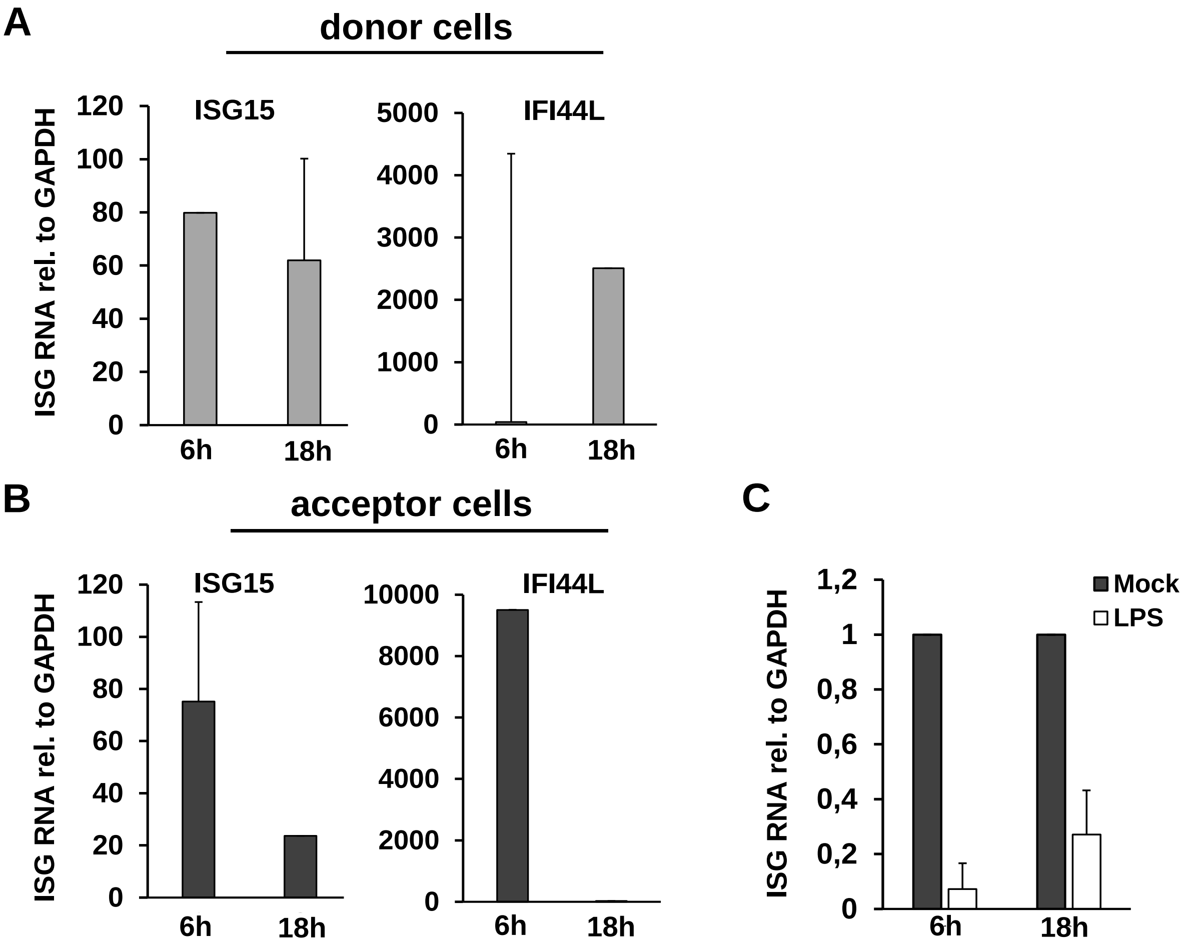

SI Figure 3: Antiviral activity of LPS-primed primary macrophages. (A) Relative levels of ISG15 and IFI44 mRNA levels in MDMs (donor cells). MDMs were stimulated with LPS (1µg/ml) for 1h, followed by washing with PBS for three times. mRNAs were quantified by real-time PCR after 6h and 18h. **(B, C)** Supernatants were collected and transferred to Huh-7.5 cells harbouring subgenomic HCV replicons (acceptor cells). ISG levels (B) and HCV RNA levels (C) in acceptor hepatoma cells were quantified after 24h of exposure to THP-1 supernatants. Mean and SEM are shown of three independent experiments.

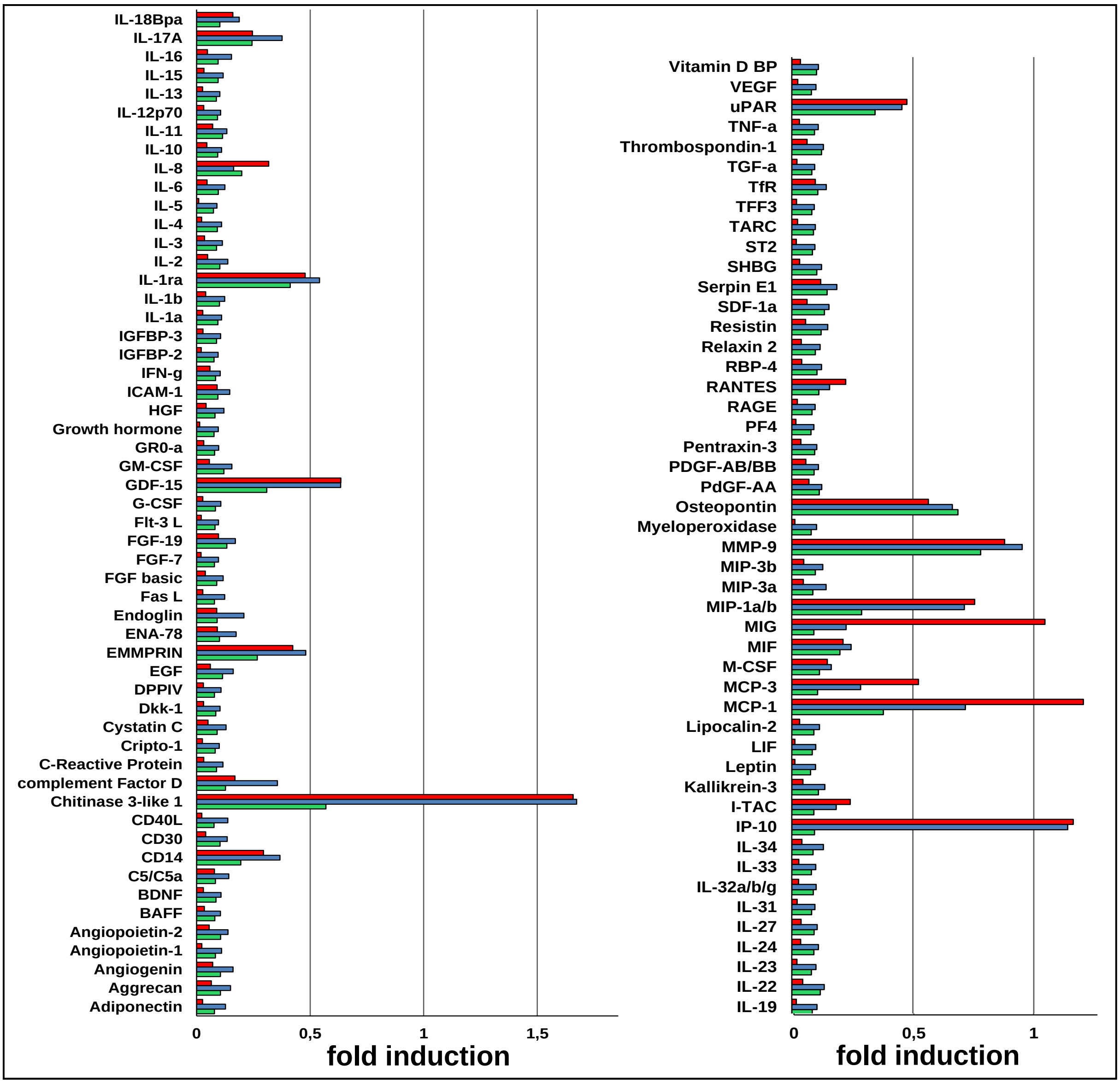

SI Figure 4: Interferon-pulsed macrophages rapidly secrete a bunch of soluble mediators. MDMs were treated with mock (green bar), IFN-α (500IU/ml, blue bar) or IFN-γ (25ng/ml, red bar) for 1h. Cytokine levels in supernatants were assessed after 8h using the Proteome Profiler Human XL Cytokine Array Kit (R&D Systems).

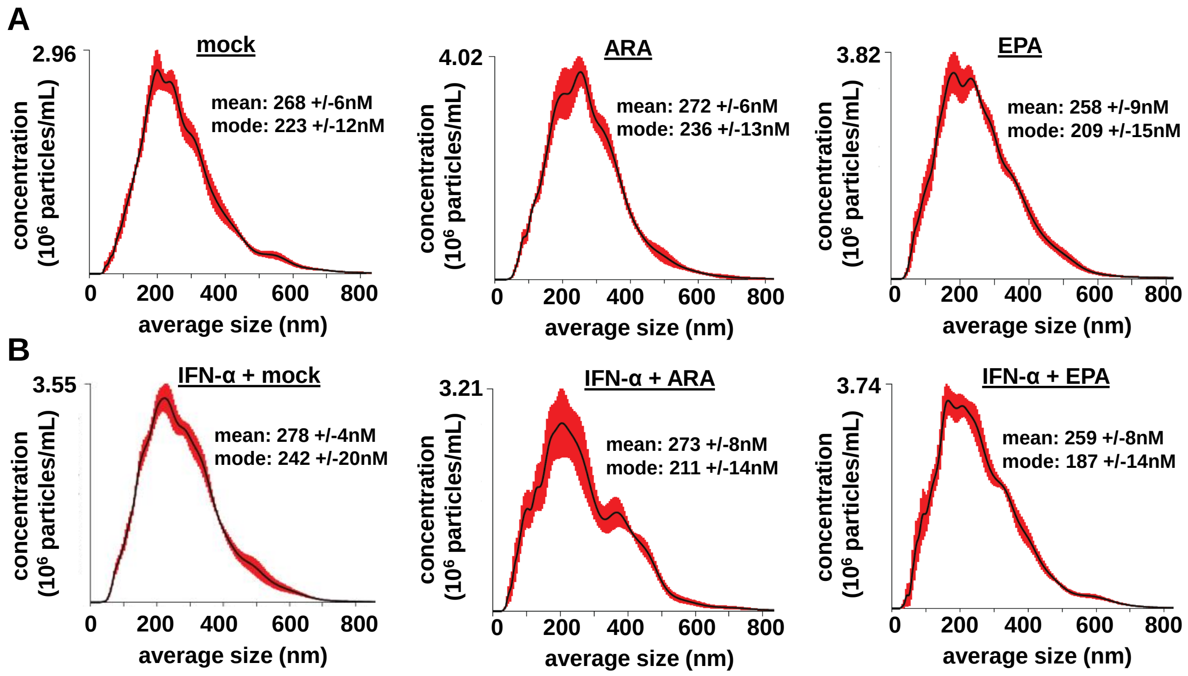

SI Figure 5: Extracellular vesicles concentration and size distribution analysis. Number of EV particles per ml as well as mean and mode size of EVs were assessed by Nano-Sight analysis. **(A)** EVs from mock, ARA or EPA treated THP-1 cells. **(B)** EVs from IFN-α pulsed THP-1 cells pre-treated with mock, ARA or EPA for 48h. Red error bars indicate SEM of six technical replicates.
